# A Comparative Study of Knowledge, Attitude, and Determinants of Tuberculosis-Associated Stigma in Rural and Urban Communities of Lagos State, Nigeria

**DOI:** 10.1155/2020/1964759

**Published:** 2020-12-03

**Authors:** David A. Oladele, Mobolanle R. Balogun, Kofoworola Odeyemi, Babatunde L. Salako

**Affiliations:** ^1^Clinical Sciences Department, Nigerian Institute of Medical Research, Lagos, Nigeria; ^2^Department of Community Medicine and Primary Care, College of Medicine, University of Lagos, Lagos, Nigeria

## Abstract

**Background:**

Tuberculosis (TB) is an important public health concern in Nigeria. TB-associated stigma could lead to delayed diagnosis and care, treatment default, and multidrug resistance. Understanding of TB-associated stigma is therefore important for TB control. The study is aimed at determining and comparing the knowledge, attitude, and determinants of TB-associated stigma. *Methodology*. This was a comparative cross-sectional study among adults in urban and rural areas of Lagos State, Nigeria. Respondents were selected through a multistage sampling technique and interviewed using a semistructured questionnaire, which contained the Explanatory Model Interviewed Catalogue (EMIC) stigma scale. IBM SPSS Statistics Software package version 20 was used for analysis.

**Results:**

A total of 790 respondents were interviewed. High proportions of respondents in rural and urban areas were aware of TB (97.5% and 99.2%, respectively). Respondents in the urban areas had overall better knowledge of TB compared to the rural areas (59.4% vs. 23%; *p* < 0.001), while respondents in the rural areas had a better attitude to TB (60.5% vs. 49.9%; *p* = 0.002). The majority of respondents in rural and urban areas had TB-associated stigma (93% and 95.7%, respectively). The mean stigma score was higher in the urban compared to rural areas (17.43 ± 6.012 and 16.54 ± 6.324, respectively, *p* = 0.046). Marital status and ethnicity were the predictors of TB-associated stigma in the rural communities (AOR-0.257; CI-0.086-0.761; *p* = 0.014 and AOR–3.09; CI-1.087-8.812; *p* = 0.034, respectively), while average monthly income and age of respondents were the predictors of TB-associated stigma in urban areas (AOR–0.274; CI–0.009-0.807; *p* = 0.019 and AOR-0.212; CI–0.057-0.788; *p* = 0.021, respectively).

**Conclusion:**

TB-associated stigma is prevalent in both rural and urban areas in this study. There is therefore a need to disseminate health appropriate information through the involvement of the community. Also, innovative stigma reduction activities are urgently needed.

## 1. Introduction

Tuberculosis (TB) is a curable and preventable disease [1]. It is the ninth leading cause of death worldwide and the leading cause of death from a single infectious agent [2, 3]. Nigeria is among the 30 high TB burden countries according to the World Health Organization (WHO). It is also among the eight countries that accounted for two-thirds of the global burden of TB in 2018 [4]. In order to sustain the global fight to end TB, the WHO proposed a paradigm shift in TB control through the “End TB strategy” program [5]. This Global Plan also identifies eight fundamental changes that must be implemented as part of the paradigm shift needed to end TB [5]. One of these changes recommends that countries should prohibit stigma and discrimination against people infected with TB [5].

Health-related stigma could be defined as a “social process that exists when elements of labeling, stereotyping, separation, loss of status, and discrimination occur in a power situation that allows them” [6]. Health-related stigma is known to cause psychological suffering, shame, and isolation in afflicted individuals and families; it could compromise the efforts to provide effective health care and could foster discrimination in societies. Stigma may also directly affect the course and outcome of the stigmatized medical condition [6]. The stigma associated with TB is the least studied among health-related stigmas when compared to other infectious diseases like leprosy and HIV/AIDS [6–9].

The prevalence of stigma associated with TB has been shown to vary from 27% to as high as 82% in different settings across the world [10–13]. It was also found to be associated with nonadherence to treatment and treatment failure [14]; it hampers investigations of household contacts [15] and causes delay in case detection [16] and also associated with high mortality due to late presentation [17]. A study of the trend of TB case notification in Lagos state between 2011 and 2015 showed a decrease in the Case Notification Ratio from 82.9% in 2013 to 72.1% in 2015 [18]. The gap between TB incidence and case notification in Nigeria is 12% [4], making Nigeria one of the 10 countries that accounted for 80% of the estimated global gap between incidence and case notification. This gap is mainly due to underreporting and underdiagnosis, which could result directly or indirectly from TB-associated stigma [4].

Therefore, eradicating the stigma surrounding TB is therefore a crucial component of efforts aimed at encouraging people to seek early diagnosis and treatment, to adhere to treatment guidelines and treatment completion along with psychosocial support or social protection benefits after diagnosis [6–8].

Furthermore, the health-related stigma associated with infectious diseases has been shown to vary among rural and urban dwellings as well as with the knowledge and educational background of the people [19]. For instance, the stigma associated with leprosy was said to differ significantly between rural and urban dwellers [20, 21], and a rural-urban dichotomy has been documented to influence the experience of HIV felt stigma [22, 23]. Also, a systematic review has documented the presence of cultural variations in knowledge, attitudes, and health responses to TB stigma [24]. However, there is a paucity of evidence in the literature comparing the stigma associated with TB among rural and urban dwellers in Nigeria.

The study, therefore, is aimed at assessing and comparing the knowledge, attitude, and determinants of the stigma associated with TB between rural and urban dwellers in Lagos, Nigeria. Understanding of this may lead to improved quality of care of TB patients in rural and urban areas in Nigeria as well as improved health and wellbeing of the general population.

## 2. Methods

### 2.1. Study Setting

The study was conducted in Lagos State, which is located in the southwestern part of Nigeria. The state accounts for over five percent of the national estimated population [25]. It has an estimated population of 23,305,971 by 2015 projection [26]. Lagos State is divided into 20 Local Government Areas (LGAs) of which 16 comprise the urban LGAs and the remaining four LGAs are classified as rural LGAs. The study took place at Lagos Mainland LGA (Urban) and Badagry LGA (Rural).

### 2.2. Study Design and Population

The study was a comparative cross-sectional study, and the respondents were adults 18 years and above in the urban and rural areas in Lagos state. Adult community members that had lived in the community for at least 6 months in the 12 months period preceding the interview were included in the study while those who had been diagnosed with TB or currently on TB treatment were excluded.

### 2.3. Sample Size Determination

The sample size for the study was determined by using the formulae for comparison of proportions [27]:

(1) $$\begin{array}{c} n=\frac{{\left(\text{Z}\alpha +\text{Z}\beta \right)}^{2}\left\{\text{P}1\left(1-\text{P}1\right)+\text{P}2\left(1-\text{P}2\right)\right\}}{{\left(\text{P}1-\text{P}2\right)}^{2}}. \end{array}$$

After adjusting for a 10% nonresponse rate, the final sample size was 380 for each of the rural and urban communities was used.

### 2.4. Sampling Methodology

A multistage sampling technique was used for respondents' selection. In stage one, 2 LGAs were selected by simple random sampling. Stage two involved the selection of 2 wards each from the urban and rural LGAs through simple random sampling. In stage three, 10 streets were randomly selected from each of the selected wards in the urban and rural communities, and in stage four, 20 houses were selected on each street. The habitable houses in each street or settlement were counted and the total number divided by 20 to obtain the sampling interval. The first house on each street was selected by balloting, and the subsequent *n*^th^ house was sampled until 20 houses had been selected in each of the 20 streets or settlements. In stage five, households for sampling were chosen from the houses selected. In each house, when more than one household exists, a list of all the households was created, and one household was selected by balloting. However, whenever only one household is present in the selected house, the household was selected. When a selected house had no eligible respondent, then the next house was used.

### 2.5. Data Collection Tool and Technique

A pretested, interviewer-administered questionnaire was used to collect data from house to house between July and October 2017. The study also makes use of a stigma scale, which has been validated to assess health-related stigma for various diseases including TB [28, 29].

The questionnaire was divided into four sections: Section A focused on the sociodemographic characteristics of the respondents and their household. Section B focused on the respondents' knowledge about TB cause, mode of transmission, prevention, and care adapted from WHO advocacy, communication, and social mobilization for TB control: a guide to developing knowledge, attitude, and practice surveys [30]. Section C focused on the respondents' attitude to TB. A three-point Likert scale that assessed the respondent's attitude with regards to susceptibility to TB infection, the severity of TB infection, benefits of TB treatment, and barriers to TB treatment was used [31]. Section D contained the adaptation of the Explanatory Model Interview Catalogue (EMIC) stigma scale for the community [28, 29]. It was designed to evaluate the potential stigmatizers in the community. It assesses perceived stigma and the attitudes of community members towards persons affected with TB. The outcome was determined by summing up the scores per each question. The higher the score, the more the likelihood of TB associated stigma.

### 2.6. Pretesting and Validation of Study Instrument

The questionnaire was pretested among 30 adults (18 years and above) each in urban and rural communities similar to the study sites. The internal consistency of the EMIC scale was assessed using Cronbach's alpha test, and a score of 0.87 was obtained.

### 2.7. Data Management

Data entry and analysis were done using the IBM Statistical Package for the Social Sciences (SPSS) Version 20.0 (IBM Corp, Armonk, NY). Knowledge of TB was categorized as good versus poor knowledge by evaluating respondents with appropriate knowledge of TB. Appropriate knowledge of TB was defined as the ability to recognize 5 knowledge domains as follows: able to recognize germ/bacteria as the cause of TB, know that TB is spread through the air, know that cough more than 2 weeks a major symptom, prevention by covering mouth, and treated by modern medicine. Individuals who recognized the 5 domains were said to have good knowledge, and those who recognize less than 5 were said to have poor knowledge [32]. Questions on attitude were scored on a 3-point scale graded 1-3 [31], and the minimum and maximum scores obtainable were 15 and 45, respectively. A score below the median (39) was regarded as a negative attitude, and an attitude score above the median was termed a positive attitude.

The total numbers of questions for the EMIC stigma scale were 15. It has the answer options (score) as follows: yes (2), possibly (1), do not know (0), and no (0). The cut-off point of “8” on the EMIC stigma scale was used to assess the stigma associated with TB. Respondents were considered to have existing stigmatization when they answered at least 4 questions with “yes,” or 8 questions with “possibly,” or the combination of both answers with a sum score at 8. A score of 8 was chosen to increase the specificity of the cut-off point and to avoid false positives [29].

The scores from the tools were categorized into dichotomous variables. Discrete variables were presented as percentages, while continuous variables were expressed as means (± standard deviation). Proportions for categorical variables were compared using Pearson Chi-square, while the difference in means was compared using the Students t-test. The level of significance was predetermined at *p* < 0.05. Variables attaining significance were further subjected to multivariable analysis using simple and multiple logistic regression to determine the predictors for “TB associated stigma.” Results were expressed as odds ratios with 95% confidence intervals.

### 2.8. Ethical Considerations

The Health Research and Ethics Committee of the Lagos University Teaching Hospital (LUTH), Lagos, Nigeria (ethical approval number: ADM/DCST/HREC/1282), and Institutional Review Board of Nigerian Institute of Medical Research, Lagos, Nigeria (ethical approval number: IRB/17/029) reviewed the protocol, the associated informed consent forms, and study-related documents and gave ethical approval. Permission was also obtained from the local government authority and the Ward Health Committee of the selected wards for the community interview. Written informed consent was obtained from each study participant prior to enrolment in the study. All staff involved in this study had appropriate training on research ethics emphasizing the importance of informed consent and confidentiality. Adequate privacy was ensured during study procedures, and effort was made to protect participants' confidentiality to the extent possible. Respondents were also informed of their right to opt-out of the study if they choose to at any point in the study.

## 3. Results

A total of 790 respondents were interviewed: three hundred and ninety-five (395) respondents in Mainland LGA and another 395 respondents in Badagry LGA of Lagos state.

The mean age of respondents in the rural and urban communities was 32 ± 11.09 years and 36.2 ± 12.58 years, respectively, with the mean age of urban areas significantly higher than rural areas (*p* < 0.05). The other study participants' demographic characteristics are as shown in Table 1.

More than four-fifth 385 (97.5%) of respondents in the rural area had ever heard about TB compared to 392 (99.2%) of respondents in the urban area. However, this difference was not statistically significant. The common sources of information in these settings were radio, television, healthcare worker, family members, and friends. Significantly higher proportions of respondents in the urban areas got information about TB from radio, television, and healthcare workers when compared to rural areas (Table 2).

A significantly higher proportion of urban respondents 233(59.4%) had good knowledge of TB when compared to rural areas 88 (23%) (*p* < 0.001). Also, respondents with good knowledge of TB were about five times more in urban areas compared to rural areas (OR: 4.896; CI: 3.584-6.689) (Table 3).

An assessment of the attitude of respondents to TB revealed that more respondents in rural areas 233 (60.5%) had a positive attitude score towards TB when compared to urban area 196 (49.9%) (*p* = 0.002) (Table 4).

Assessing the stigma in the community using the EMIC scale showed that a greater proportion of respondents in the urban area (27.6%) felt a person infected with TB would like to keep others from knowing they have TB if possible compared to (20.3%) in the rural area (*p* = 0.001). More respondents in the rural area would think less of themselves if a family member is infected with TB (*p* = 0.036). More respondents from the urban area (41.1%) than the rural area (28.2%) felt that knowing that someone has TB would adversely affect other family members (*p* = 0.001), and a higher proportion of respondents in both rural and urban areas felt people in the community would avoid a person with TB just as a higher proportion of urban respondents (52.3%) than rural respondents (43.3%) would likely refuse to visit the home of a person infected with TB (*p* = 0.042).

About 46.5% of urban respondents compared to rural respondents (30.6%) will have concerns about disclosure if one member of their family is infected with TB (*p* < 0.001).

TB was believed could constitute a problem of preventing a person infected in getting married (45.3%) in rural areas compared to urban areas (39.5%) (*p* < 0.001) but more of the respondents form urban area (31.9%) felt that TB could cause a problem in an ongoing marriage and also cause a problem for a relative of TB patient to get married (*p* < 0.001).

A higher proportion of respondents in the rural areas (88.3%) and urban areas (87.5) would not like to buy food from a person affected by TB (*p* = 0.035) (Table 5).

In all, more respondents 375 (95.7%) in the urban area had TB-associated stigma when compared to rural area 357 (93.0%), and this difference was not statistically significant (*p* = 0.071). The mean EMIC scale score for the urban area is 17.45 ± 6.007, which is significantly higher than that of the rural area 16.55 ± 6.317 (Student *t*-test = −2.00; *p* = 0.046) (Table 6).

Ethnicity was found to have a statistically significant association with TB-associated stigma in rural and urban communities. Respondents belonging to minority ethnic groups (particularly Egun ethnicity) in the rural areas had a significant proportion of perceived TB-associated stigma compared to the urban areas (Table 7).

Respondents with good knowledge (97.4%) of TB had a higher proportion of TB-associated stigma when compared to those with poor TB knowledge (93.1%) in the urban communities. This difference was found to be statistically significant (*p* = 0.038) (Table 8).

Multivariable analysis to assess the predictors of TB-associated stigma revealed that in the rural areas, ethnicity (*p* value = 0.014) and marital status (*p* value = 0.034) predicted TB-associated stigma. Thus, there was a 74.3% reduced likelihood of TB-associated stigma among non-Yoruba people as compared to the Yorubas, (AOR = 0.257, 95% CI: 0.0865-0.761, *p* value = 0.014), Also, there was a 3-fold odds of TB-associated stigma among unmarried respondents as compared to the married ones (AOR = 3.096, 95% CI: 1.087-8.812, *p* value = 0.034) (Table 9).

In the urban area, average monthly income (*p* = 0.019) and age of respondents (*p* = 0.021) predicted TB-associated stigma. There was a 72.6% reduced likelihood of TB-associated stigma among individual earning less than $50 monthly as compared to those earning more than $50, (AOR = 0.274, 95% CI: 0.093-0.807, *p* value = 0.019), Also, there was a 78.8% reduced likelihood of TB-associated stigma among individuals less than 35 years old as compared to those older than 35 years (AOR = 0.212, 95% CI: 0.057-0.788, *p* value = 0.021) (Table 10).

## 4. Discussion

In this study, 790 adults were interviewed to determine knowledge, attitude, and determinants of TB-associated stigma in rural and urban communities in Lagos State.

Our study is the first study to the best of our knowledge that has examined TB-associated stigma and its disparities in rural and urban communities in Nigeria.

TB is not an unknown illness in communities studied as the majority of respondents in rural and urban areas (97.5% and 99.2%, respectively) had ever heard about the disease. This could be due to the good health information and public enlightenment program in this part of Nigeria. This finding is comparable to that of studies from Libya and Ethiopia that reported 96.5% and 94.9% awareness of TB in the community, respectively [33, 34]; it is also similar to a local study from Ogoja in Cross River State, Nigeria, that reported TB awareness of 97% in the study population [35]. Our finding on awareness of TB in the community was however higher than other Nigerian studies: 93% in Enugu state [36] and 86.5% in Edo state [37].

Our study also revealed a better appropriate knowledge of TB in urban areas compared to rural areas using the respondent's ability to respond to 5 cardinal knowledge questions (*p* < 0.001). This is comparable to a study among prisoners in Ethiopia in which those in urban areas had better overall knowledge compared to those in rural areas [32, 38]. The respondents in rural communities had a better overall positive attitude compared to urban areas but there was no statistically significant association between attitude and sociodemographic factors in the rural and urban communities.

An evaluation of the specific items on the EMIC stigma scale revealed that a higher proportion of urban respondents (27.6%) believed that a person who has TB would desire to keep others from knowing if possible compared to 20.3% among rural respondents (*p* < 0.001). This could be because communal living in rural areas could limit the ability to conceal the disease in this area compared to the urban areas. This could influence the rate of TB diagnosis in these communities as well as adherence to medication. However, the proportion of participants who will desire to conceal TB diagnosis in our study is higher than what was reported from a previous study in rural Ethiopia but lower than 75% reported from a study in rural India [39] and 37.3% reported from another study from Thailand [29].

Also, more respondents in the rural area 17.6% compared to urban area 11.5% would feel inferior to other community members if someone in their family had TB. This could be due to the fact that most people in rural know each other because of living in close proximity compared to an independent lifestyle that is characteristics of urban areas of Lagos metropolis. This finding is similar to a study from Thailand that reported that 17.4% of respondents would think less of themselves if a family member had a TB diagnosis [39]. But lower than the findings of a study from the Indian rural community with 85% rural dwellers who would have diminished self-esteem if a family member was infected with TB [29].

This present study also corroborated the fact that TB infection could cause shame and embarrassment in the community as reported by respondents from rural 45.2% and urban 48.2% communities. This is similar to 48% reported from a study in rural India [39], but higher than 20.3% reported from Thailand [29], 31% reported among prisoners in Ethiopia [37], and 39% reported from a community study in Ethiopia [40]. However, it is lower than 76.3% reported among community respondents in Pakistan that believe TB causes shame [41].

Another stigmatizing behavior in the community to TB patients is that persons in the community usually look down on them. An equal proportion of respondents in rural 37.6% and urban 37.5% areas alluded to this behavior. This finding is however lower than 51% reported among rural dwellers in Ethiopia [40] but higher than that of Thailand study that reported that 22.5% of community members would think less of TB patients [29].

Just like it was reported for other infectious diseases like leprosy, about half of the rural 48.6% and urban 53.1% of respondents believed that people in their communities would avoid a person infected with TB. This is mainly due to the fact that respondents were afraid of contracting the infection if they associate closely with community members who are infected with TB. Also, rural respondents in this study believed that TB infection could reduce the prospect of getting marital partners compared to urban respondents (*p* < 0.001). This could be because people in communities in rural areas hold on to more traditional values in their approach to choosing a marital partner to compare to people living in urban areas. This also could be a reflection of the cultural practice prevalent in our setting in which diseases are perceived to be hereditary, and families do not want to be associated with individuals with chronic diseases [42]. The proportion of those reporting TB could reduce the prospect of finding a marital partner is higher than 20% reported rural Ethiopian study [40] and 30.1% reported from Thailand [29].

TB was also believed to also cause problems in existing marriages. About 31.9% of respondents in urban areas alluded to this when compared to 15.3% among the respondents from the rural areas (*p* < 0.001). This could be due to the fact that persons living in rural areas are more cohesive and likely to be engaged in family and communal support than individuals living in urban areas. However, TB has been reported as a possible cause of marital difficulty in other studies too. For instance, the study of cultural epidemiology of TB in Bangladesh, India, and Malawi reported that divorce and isolation could occur in marriage due to TB infection [43]. Also, another study from Thailand reported that 22.5% of respondents believed that TB could cause a problem in an ongoing marriage [29].

A significantly higher proportion of respondents from rural areas 81.6% believed that TB could cause difficulty in finding a job and earning a living compared to 69.4% among urban respondents (*p* < 0.001). This could be due to the fact that potential employers might avoid the persons infected with TB due to fear of being infected. However, our findings on the difficulty of getting a job for a person infected with TB are higher than 34.75% reported from a Thailand study. The implication of this will include poor economic power and the extreme difficulty of persons infected with TB to manage the catastrophic direct and indirect cost of treating the illness [44].

Generally, the majority of respondents in our study could dislike buying food from a person affected by TB (rural 88.3% and urban 87.3%). This could be because members of communities in these areas have the fear that they can be infected with TB through the food they buy. This finding is however higher than 41.5% reported from a Thailand study [29]. This finding could also be a reflection of the cultural practice in the study settings in which there must be societal acceptance of a person selling food before their food is considered fit for consumption.

Overall, our study reported a high prevalence of TB-associated stigma in the communities studied, and the majority of respondents in the rural areas (93%) and urban areas (95.7%) had TB-associated stigma. However, the mean EMIC stigma scale score is higher among the urban respondents 17.43 ± 6.012 compared to rural respondents 16.54 ± 6.324, and the difference is statistically significant (Student *t*-test-2, *p* = 0.046). However, the proportion of TB-associated stigma in our study is higher than those reported by different previous studies. A study in India reported a 73% prevalence of stigmatizing attitudes in the community [11], and another study from Pakistan believed that 47.9% of TB patients are stigmatized [41]. Also, a study in the rural community of Ethiopia reported 51.2% had stigma [40], and another study among the pastoralist population in Ethiopia reported 61.8% perceived TB-associated stigma [45] just as a study of Tunisia reported 54.9% of TB-associated stigma [46]. The high proportion of respondents with perceived TB-associated stigma in this study could be due to diverse cultural practices and lack of adequate and correct health information on TB [24].

A major determinant of TB-associated stigma in the rural community was ethnicity and marital status. Respondents who belong to ethnic groups other than the Yoruba tribe had about 74% reduced likelihood of having TB-associated stigma in the community when compared to those of Yoruba ethnicity. Also, unmarried individuals had about 3 times the odds of TB-associated stigma when compared to married individuals. There might be a need to target stigma reduction activities at unmarried individuals in the rural communities because the social support provided by marriage could have contributed to the reduced odds of TB-associated stigma in the rural community.

However, good knowledge of TB, age ≥ 35 years, and monthly income ≥ $50 were found to be associated with TB-associated stigma in the urban community in bivariate analysis. After adjusting for confounders, however, individuals earning more than $50 had about 4 times the odds of TB-associated stigma. So also, individuals who are less than 35 years had about 79% reduced likelihood of TB-associated stigma in the community compared to respondents older than 35 years. This finding is similar to a study in southern Thailand that reported that older age is associated with TB stigma [39]. It is also similar to a community study in India [11] and Pakistan [41] that reported that high-income groups have a higher chance of TB-associated stigma than low-income earners. However, this present study did not find any relationship between gender and TB-associated stigma in contrast to a previous report by the study of the explanatory model of TB stigma in Bangladesh, India, Columbia, and Malawi [43].

An important recommendation to mitigate the negative effect of TB-associated stigma on TB control in Nigeria is that the Ministry of Health at the national and subnational level in line with WHO End TB Strategy should strengthen the stigma reduction component of the existing National Strategic Plan for Tuberculosis Control (2015-2020) that focuses on improving universal access to TB treatment through the creation of a supportive environment, free of stigma and discrimination [47].

A major limitation to generalizing the outcome of the study could be because it was conducted in rural and urban communities of Lagos State, Nigeria. The state is a cosmopolitan state, which has better healthcare indices, compared to other states in Nigeria with arguably better health coverage and better healthcare provider to patient's ratio. Hence, the findings of this study could be different from other states in Nigeria. However, a further study with participants recruited from the six geopolitical zones of Nigeria will provide nationally representative data.

## 5. Conclusion

There is a high prevalence of TB-associated stigma in rural and urban communities in this study. Ethnicity and marital status were found to be determinants in the rural community while age ≥ 35 years and average monthly income ≥ $50 were found to be determinants of TB-associated stigma in urban communities. There is therefore a need to increase access to appropriate information on TB and TB-associated stigma through print, radio, television, and social media. This information should be age-appropriate and culturally acceptable in local languages. Also, further research into stigma reduction intervention measures will help to provide insight into the appropriate public health approach aimed at mitigating the impact of TB-associated stigma to the national TB control program.

## Figures and Tables

**Table 1:** Sociodemographic characteristics of respondents.

Variable	Rural (*n* = 395) Freq. (%)	Urban (*n* = 395) Freq. (%)	Total (*n* = 790)	*χ* ^2^	df	*p* value
Age						
<25	113 (28.6)	76 (19.2)	189 (23.9)	27.04	5	<0.001^∗^
25-34	142 (35.9)	113 (28.6)	255 (32.3)			
35-44	90 (22.8)	115 (29.1)	205 (25.9)			
45-54	29 (7.3)	57 (14.4)	86 (10.9)			
55-64	15 (3.8)	19 (4,8)	34 (4.3)			
≥65	6 (1.5)	15 (3.8)	21 (2.7)			
Mean age	32 ± 11.079	36.2 ± 12.576		*t* = −4.139		<0.05^∗^
Sex						
Male	207 (52.4)	192 (48.6)	399 (50.5)	1.139	1	0.160
Female	188 (47.6)	203 (51.4)	391 (49.5)			
Ethnicity (*n* = 783)						
Yoruba	147 (37.2)	295 (76.4)	442 (56.6)	284.0	3	<0.001^∗^
Igbo	34 (8.6)	73 (18.9)	107 (13.7)			
Hausa	1 (0.3)	16 (4.1)	17 (2.2)			
Others^∗∗^	213 (53.9)	2 (0.5)	215 (27.5)			
Religion						
Christianity	295 (74.7)	244 (61.8)	539 (68.2)	16.96	2	<0.001^∗^
Islam	96 (24.3)	149 (37.7)	246 (31.0)			
Others	4 (1.0)	2 (0.5)	6 (0.8)			
Marital status						
Married	261 (66.1)	240 (60.8)	501 (63.4)	11.0	3	<0.001^∗^
Divorced/separated	6 (1.5)	23 (5.8)	29 (3.7)			
Widowed	13 (3.3)	16 (3.8)	28 (3.5)			
Single	115 (29.1)	117 (29.6)	232 (29.4)			
Education status						
No formal education	51 (12.9)	17 (4.3)	69 (8.6)	61.89	3	<0.001^∗^
Primary	97 (24.6)	52 (13.2)	149 (18.9)			
Secondary	208 (52.7)	220 (55.7)	428 (54.2)			
Tertiary	39 (9.9)	107 (26.8)	146 (18.4)			
Employment status						
Employed	363 (91.9)	322 (81.9)	685 (86.9)	20.02	3	<0.001^∗^
Unemployed	31 (8.1)	71(18.1)	102 (13.1)			
Occupation						
Professional/managerial	14 (3.9)	55 (16.9)	69 (10.0)	63.24	4	<0.001^∗^
Clerical	6 (1.7)	11 (3.4)	17 (2.5)			
Sales and services	136 (37.6)	119 (36.5)	255 (37.1)			
Skilled manual	160 (44.2)	72 (22.1)	232 (33.7)			
Unskilled manual	46 (12.7)	69 (21.2)	115 (16.7)			
Income (naira) (*n* = 657)						
<18,000	180 (51.4)	66 (21.4)	246 (37.4)	64.66	2	<0.001^∗^
18000-30000	61 (17.4)	72 (23.4)	133 (20.2)			
>30000	109 (31.1)	170 (55.2)	279 (42.4)			
Mean income	23,057 ± 20,209	41,516 ± 41,110	*t* = −7.44; *p* = 0.010^∗^			
Median income (range)	15000 (1000 to 150000)	30000 (3000 to 400000)				
Type of house (*n* = 775)						
Duplex	4 (1.03)	1 (0.26)	5 (0.64)	45.05	4	<0.001^∗^
3/2 bedroom flat	37 (9.5)	67 (17.3)	104 (13.4)			
Self-contained	77 (19.8)	137 (35.4)	214 (27.6)			
One room shared	270 (69.6)	182 (47.0)	452 (58.3)			
Head of household						
Yes	192 (49.2)	172 (43.8)	419 (53.5)	3.25	2	0.197
No	198 (50.8)	221 (56.2)	363 (46.4)			

**Table 2:** Awareness of tuberculosis and source of information about TB.

Variable	Rural *n* = 395 Freq. (%)	Urban *n* = 395 Freq. (%)	Total *n* = 790 Freq. (%)	*χ* ^2^	*p* value
Ever heard about tuberculosis.					
Yes	385 (97.5)	392 (99.2)	779 (98.6)	2.305	0.112
No	10 (2.5)	3 (0.8)	11 (1.4)		

Source of information^	*n* = 385	*n* = 392			
Family and friends	227 (59.0)	251 (64.0)	479 (61.5)	2.15	0.082
Radio	200 (51.9)	253 (64.5)	455 (58.4)	12.22	0.001^∗^
Television	117 (30.4)	247 (63.0)	365 (46.9)	82.70	0.001^∗^
Healthcare workers	103 (26.8)	229 (58.4)	333 (42.7)	79.18	0.001^∗^
Brochures and posters	20 (5.2)	92(23.5)	112 (14.4)	52.99	0.001^∗^
Religious leaders	39 (10.1)	69 (17.6)	108 (13.9)	9.23	0.002^∗^
Newspapers and magazines	9 (2.3)	89 (22.7)	98 (12.6)	73.54	0.001^∗^
Billboards	14 (3.6)	71 (18.1)	85 (10.9)	42.0	0.001^∗^
Teachers	13 (3.4)	22 (5.6)	35 (4.5)	2.30	0.082

^∗^Multiple responses allowed.

**Table 3:** Participants appropriate knowledge of tuberculosis.

	Good knowledge Freq. (%)	Poor knowledge Freq. (%)	*χ* ^2^	df	*p* value
Rural area (*N* = 385)	88 (23.0)	294 (77.0)	105.62	1	<0.001^∗^
Urban area (*N* = 393)	233 (59.4)	159 (40.6)			
Odds ratio	4.896 (CI: 3.584-6.689)				

**Table 4:** Attitude scores of respondents to tuberculosis infection.

Attitude	Positive attitude Freq. (%)	Negative attitude Freq. (%)	*χ* ^2^	df	*p* value
Rural area (*n* = 385)	233 (60.5)	152 (39.5)	8.912	1	0.002^∗^
Urban area (*n* = 393)	196 (49.9)	197 (50.1)			

Positive attitude ≥ median attitude score of 39; Negative attitude < median attitude score of 39. ^∗^Statistical significance.

**Table:** (a) Assessment of respondent's perceived stigma to TB in the community (EMIC items 1-8)

Variables	Rural *n* = 385 Freq. (%)	Urban *n* = 392 Freq. (%)	Total *n* = 778	*χ* ^2^	*p* value
A person with TB will keep others from knowing, if possible.					
No/do not know	265 (68.7)	191 (48.7)	456 (58.6)	36.05	<0.00
Possibly	42 (10.9)	93 (23.7)	135 (17.4)		1^∗^
Yes	78 (20.3)	108 (27.6)	186 (23.9)		
Would think less of self if a family member had TB because of this person's problem.					
No/do not know	240 (62.2)	272 (69.4)	512 (65.8)	6.65	0.036^∗^
Possibly	77 (20.0)	75 (19.1)	152 (19.6)		
Yes	68 (17.6)	45 (11.5)	113 (14.5)		
TB cause shame or embarrassment					
No/do not know	82 (21.2)	85 (21.7)	167 (21.5)	2.18	0.534
Possibly	129 (33.4)	118 (30.1)	247 (31.7)		
Yes	174 (45.2)	189 (48.2)	362 (46.6)		
Others would think less of a person with TB					
No/do not know	91 (23.6)	104 (26.5)	195 (25.1)	2.055	0.561
Possibly	149 (38.6)	141 (36.0)	290 (37.3)		
Yes	145 (37.6)	147 (37.5)	292 (37.5)		
Knowing that someone has TB would have an adverse effect on others					
No/do not know	110 (28.5)	98 (25.0)	208 (26.7)	14.29	0.001^∗^
Possibly	166 (43.1)	133 (33.9)	299 (38.5)		
Yes	109 (28.2)	161 (41.1)	270 (34.7)		
Other people in this community would avoid a person affected by TB					
No/do not know	45 (11.7)	54 (13.8)	99 (12.7)	3.74	0.154
Possibly	153 (39.6)	130 (33.2)	283 (36.4)		
Yes	187 (48.6)	208 (53.1)	395 (50.8)		
People would refuse to visit the home of a person affected by TB					
No/do not know	56 (14.5)	45 (11.5)	101 (13.0)	6.33	0.042^∗^
Possibly	162 (42.0)	142 (36.2)	304 (39.1)		
Yes	167 (43.3)	205 (52.3)	371 (47.7)		
People in this community would think less of the family of a person with TB					
No/do not know	132 (34.3)	122 (31.1)	254 (32.7)	1.85	0.605
Possibly	129 (33.4)	139 (35.5)	268 (34.4)		
Yes	124 (32.1)	131 (33.5)	255 (32.7)		

^∗^Statistical significance.

**Table:** (b) Assessment of respondent's perceived stigma to TB in the community (EMIC items 9-15)

Variables	Rural *n* = 386 Freq. (%)	Urban *n* = 392 Freq. (%)	Total *n* = 778	*χ* ^2^	*p* value
TB would cause problems for the family					
No/do not know	81 (21.0)	95 (24.2)	176 (22.6)	5.95	0.051
Possibly	152 (39.5)	122 (31.1)	274 (35.3)		
Yes	152 (39.4)	175 (44.6)	327 (42.0)		
Family would have concern about disclosure if one of their members had TB					
No/do not know	110 (28.5)	77 (19.6)	187 (24.0)	22.03	<0.001^∗^
Possibly	157 (40.7)	133 (33.9)	290 (37.3)		
Yes	118 (30.6)	181 (46.2)	299 (38.5)		
TB could be a problem for a person to get married.					
No/do not know	68 (17.7)	120 (30.6)	188 (24.2)	17.81	<0.001^∗^
Possibly	142 (36.9)	117 (29.8)	259 (33.3)		
Yes	174 (45.3)	155 (39.5)	329 (42.2)		
TB could cause problems in an on-going marriage.					
No/do not know	226 (58.5)	129 (32.9)	355 (45.6)	55.86	<0.001^∗^
Possibly	100 (26.2)	138 (35.2)	238 (30.6)		
Yes	59 (15.3)	125 (31.9)	184 (23.7)		
TB could cause a problem for a relative of a patient to get married.					
No/do not know	182 (47.3)	161 (41.1)	343 (44.1)	13.68	0.003^∗^
Possibly	118 (30.6)	99 (25.3)	217 (27.9)		
Yes	85 (10.9)	132 (33.7)	217 (27.9)		
TB could cause difficulty for a person to find work.					
No/do not know	9 (2.3)	29 (7.4)	38 (4.9)	19.54	<0.001^∗^
Possibly	61 (16.1)	91 (23.2)	152 (19.6)		
Yes	315 (81.6)	272 (69.4)	587 (75.4)		
People could dislike buying food from a person affected by TB					
No/do not know	7 (1.8)	19 (4.8)	26 (3.3)	6.71	0.035^∗^
Possibly	38 (9.8)	29 (7.4)	67 (8.6)		
Yes	340 (88.3)	344 (87.8)	684 (88.0)		

^∗^Statistical significance.

**Table 6:** TB-associated stigma in the community.

EMIC score	TB stigma present Freq. (%)	TB stigma absent Freq. (%)	*χ* ^2^	df	*p* value
Rural area (*n* = 384)	357 (93.0)	27 (7.0)	2.63	1	0.071
Urban area (*n* = 392)	375 (95.7)	17 (4.3)			
Odds ratio	1.673 (0.896-3.122)				
Mean EMIC scale score					
Rural	16.54 ± 6.324	Students *t* test -2.00; *p* = 0.046^∗^			
Urban	17.43 ± 6.012				

Perceived stigma absent = EMIC score < 8; Perceived stigma present = EMIC score ≥ 8. ^∗^Statistical significance.

**Table:** (a) Association between sociodemographic characteristics of respondents and tuberculosis associated stigma in the communities

Variables	Rural (*n* = 384) TB stigma (%) present (*n* = 357)	Total	Urban (*n* = 392) TB stigma (%) present (*n* = 375)	Total	*χ* ^2^	*p* value
Sex						
Male	184 (91.5)	201	182 (95.8)	190	0.77	0.439
Female	173 (94.5)	183	193 (95.5)	202		
Age						
<25	104 (95.4)	109	73 (97.3)	75	6.96	0.223
25-34	129 (93.5)	138	100 (89.3)	112		
35-44	78 (88.6)	88	113 (98.3)	115		
45-54	27 (96.4)	28	56 (98.2)	57		
55-64	14 (93.3)	15	19 (100)	19		
≥65	5 (83.3)	6	15 (100)	15		
Ethnicity						
Yoruba	142 (97.3)	146	280 (95.2)	294	9.25	0.026^∗^
Igbo	29 (87.9)	33	69 (97.2)	71		
Hausa	1 (100)	1	16 (100)	16		
Others^∗∗^	185 (90.7)	204	1 (50)	2		
Religion						
Christianity	263 (92.3)	285	233 (96.3)	242	1.64	0.440
Islam	91 (95.8)	95	140 (94.6)	148		
Others	3 (75.0)	4	2 (100)	2		
Marital status						
Married	231 (91.3)	253	228 (95.4)	239	3.55	0.315
Divorced/separated	6 (100)	6	22 (95.7)	23		
Widowed	12 (100)	12	14 (100)	14		
Single	108 (95.6)	113	111 (95.7)	116		

^∗^Statistical significance.

**Table:** (b) Association between other sociodemographic characteristics of respondents and tuberculosis-associated stigma in the communities

Variables	Rural (*n* = 384) stigma to TB (%) present (n =357)	Total	Urban (*n* = 392) stigma to TB (%) present (*n* = 375)	Total	*χ* ^2^	*p* value
Education						
No formal	49 (98.0)	50	16 (94.1)	17	3.85	0.278
Primary	84 (90.3)	93	49 (94.2)	52		
Secondary	191 (94.1)	203	210 (96.3)	218		
Tertiary	33 (86.8)	38	101 (95.3)	106		
Employment						
Employed	329 (93.5)	352	313 (96.0)	326	1.24	0.249
Unemployed	28 (87.5)	32	63 (94.0)	67		
Occupation (*n* = 675)						
Professional/managerial	12 (85.7)	14	54 (98.2)	55	2.06	0.725
Clerical	6 (100)	6	11 (100)	11		
Trading	124 (93.9)	132	114 (96.6)	118		
Skilled manual	144 (94.1)	153	65 (91.5)	71		
Unskilled manual	42 (91.3)	46	67 (97.1)	69		
Type of housing						
Duplex	4 (100.0)	4	1 (100)	1	0.70	0.873
3/2 bedroom flat	34 (94.4)	36	64 (95.5)	67		
Self-contained	70 (90.9)	77	132 (97.1)	136		
One room shared	242 (93.4)	259	171 (94.5)	181		
Income amount (naira) (*n* = 647)						
<18000	165 (93.8)	176	58 (87.9)	66	2.84	0.242
18000-30000	52 (91.2)	57	68 (95.8)	71		
>30000	100 (92.6)	108	165 (97.6)	169		

^∗^Statistical significance.

**Table:** (a) Association between respondent knowledge and attitude and perceived TB-associated stigma in rural communities

Variables	Perceived TB stigma present (%)	Perceived TB stigma absent (%)	Total	*χ* ^2^	*p* value
Attitude to TB					
Positive	219 (94.4)	13 (5.6)	232	1.83	0.176
Negative	138 (90.8)	14 (9.2)	152		
Knowledge of TB					
Good	82 (94.3)	5 (5.7)	87	0.31	0.579
Poor	272 (92.5)	22 (7.5)	294		

**Table:** (b) Association between respondent knowledge and attitude and perceived TB-associated stigma in urban communities

Variables	Perceived TB stigma present (%)	Perceived TB stigma absent (%)	Total	*χ* ^2^	*p* value
Attitude to TB					
Positive	188 (95.9)	8 (4.1)	196	0.06	0.812
Negative	188 (95.4)	9 (4.6)	197		
Knowledge of TB					
Good	227 (97.4)	5 (2.6)	233	4.30	0.038^∗^
Poor	148 (93.1)	11 (6.9)	159		

^∗^Statistical significance.

**Table 9:** Simple and multiple logistic regressions of TB-associated stigma and associated factor in rural communities.

Predictor variable	Simple logistic regression				Multiple logistic regression			
	*p* value	Crude odds ratio	95% CI lower upper		*p* value	Adjusted odds ratio	95% CI lower upper	
Ethnicity								
Other ethnic groups	0.018^∗^	0.263	1.716	4.241	0.014^∗^	0.257	0.086	0.761
Yoruba		1				1		
Marital status								
Unmarried	0.085	2.4	0.887	6.491	0.034^∗^	3.09	1.087	8.812
Married		1				1		
Age								
≥35 years	0.165	1.745	0.795	3.828				
<35 years		1						
Sex								
Male	0.255	1.598	0.712	3.587				
Female		1						
Employment								
Employed	0.215	0.489	0.158	1.514				
Unemployed		1						
Attitude								
Negative	0.181	1.709	0.779	3.745				
Positive		1						

**Table 10:** Simple and multiple logistic regressions of TB-associated stigma on associated factors in urban communities.

Predictor variable	Simple logistic regression				Multiple logistic regression			
	*p* value	Crude odds ratio	95% CI lower upper		*p* value	Adjusted odds ratio	95% CI lower upper	
Income (naira)								
<18000	0.005^∗^	0.217	0.075	0.625	0.019^∗^	0.274	0.093	0.807
>18000		1				1		
Age								
<35 years	0.008^∗^	`0.182	0.052	0.645	0.021^∗^	0.212	0.057	0.788
≥35 years		1				1		
Knowledge								
Good	0.046^∗^	2.812	1.018	7.767	0.33	1.72	0.575	5.165
Poor		1				1		
Sex								
Male	0.914	0.947	0.357	2.508				
Female		1						
Ethnicity								
Yoruba	0.566	1.45	0.407	5.162				
Non-Yoruba		1						

^∗^Significant.

## Data Availability

The electronic data (in Microsoft Excel format) used to support the findings of this study may be released upon application to the Nigerian Institute of Medical Research Institutional Review Board, who can be contacted at nimr_irb@yahoo.com.

## References

[1] Maher D. (2006). The global plan to stop TB, 2006-2015. Actions for life: towards a world free of tuberculosis. *The International Journal of Tuberculosis and Lung Disease*.

[2] World Health Organization (2017). *Global Tuberculosis Report 2017*.

[3] World Health Organization (2015). *Global Tuberculosis Report 2015*.

[4] World Health Organization (2019). *Global Tuberculosis Report 2019*.

[5] Stop T. B., Partnership U. (2016). *The Paradigm Shift 2016-2020, Global Plan to End TB [Internet]*.

[6] Mak W. W., Cheung F., Woo J. (2009). A comparative study of the stigma associated with infectious diseases (SARS, AIDS, TB). *Hong Kong Medical Journal*.

[7] Hatherall B. (2009). *Understanding TB-Related Stigma in Asia. R4DDfidGovUk [Internet]*.

[8] van Rie A., Sengupta S., Pungrassami P. (2008). Measuring stigma associated with tuberculosis and HIV/AIDS in southern Thailand: exploratory and confirmatory factor analyses of two new scales. *Tropical Medicine & International Health*.

[9] Macq J., Solis A., Martinez G. Can we make TB control more pro-poor by addressing the sticky business of stigma?.

[10] Courtwright A., Turner A. N. (2010). Tuberculosis and stigmatization: pathways and interventions. *Public Health Reports*.

[11] Sagili K. D., Satyanarayana S., Chadha S. S. (2016). Is knowledge regarding tuberculosis associated with stigmatising and discriminating attitudes of general population towards tuberculosis patients? Findings from a community based survey in 30 districts of India. *PLoS One*.

[12] Ali S. S., Rabbani F., Siddiqui U. N. (2003). Tuberculosis: do we know enough? A study of patients and their families in an out-patient hospital setting in Karachi, Pakistan. *The International Journal of Tuberculosis and Lung Disease*.

[13] Cremers A. L., de Laat M. M., Kapata N., Gerrets R., Klipstein-Grobusch K., Grobusch M. P. (2015). Assessing the consequences of stigma for tuberculosis patients in urban Zambia. *PLoS One*.

[14] Duko B., Bedaso A., Ayano G., Yohannis Z. (2019). Perceived stigma and associated factors among patient with tuberculosis, Wolaita Sodo, Ethiopia: cross-sectional study. *Tuberculosis Research and Treatment*.

[15] Ngamvithayapong-Yanai J., Luangjina S., Thawthong S., Bupachat S., Imsangaun W. (2019). Stigma against tuberculosis may hinder non-household contact investigation: a qualitative study in Thailand. *Public Health Action*.

[16] Osei E., Akweongo P., Binka F. (2015). Factors associated with DELAY in diagnosis among tuberculosis patients in Hohoe Municipality, Ghana. *BMC Public Health*.

[17] Adamu A. L., Gadanya M. A., Abubakar I. S. (2017). High mortality among tuberculosis patients on treatment in Nigeria: a retrospective cohort study. *BMC Infectious Diseases*.

[18] Adejumo O. A., Daniel O. J., Abdur-Razzaq H. A. (2017). Trend of tuberculosis case notification and treatment outcome in Lagos State, Nigeria: a 5-year retrospective study. *Transactions of the Royal Society of Tropical Medicine and Hygiene*.

[19] Costelloe S., Kemppainen J., Brion J. (2015). Impact of anxiety and depressive symptoms on perceptions of stigma in persons living with HIV disease in rural versus urban North Carolina. *AIDS Care*.

[20] Naidoo J. R., Uys L. R., Greeff M. (2007). Urban and rural differences in HIV/AIDS stigma in five African countries. *African Journal of AIDS Research*.

[21] Roosta N., Black D. S., Rea T. H. (2013). A comparison of stigma among patients with leprosy in rural Tanzania and urban United States: a role for public health in dermatology. *International Journal of Dermatology*.

[22] Yebei V. N., Fortenberry J. D., Ayuku D. O. (2008). Felt stigma among people living with HIV/AIDS in rural and urban Kenya. *African Health Sciences*.

[23] Hong Y., Li X., Stanton B. (2008). Expressions of HIV-related stigma among rural-to-urban migrants in China. *AIDS Patient Care and STDs*.

[24] Chang S., Cataldo J. K. (2014). A systematic review of global cultural variations in knowledge, attitudes and health responses to tuberculosis stigma. *The International Journal of Tuberculosis and Lung Disease*.

[25] National Population Commission (NPC) (2008). Macro (2009). *National Population Commission, Nigeria. Nigerian Demographic and Health Survey*.

[26] Lagos Bureau of Statistic (2015). *Lagos State Government Digest of Statistics 2015*.

[27] Prabhakara G. *Biostatistics*.

[28] van Brakel W., Voorend C., Ebenso B., Cross H., Augustine V. Guidelines to reduce stigma [Internet]. *How to Assess Health-Related Stigma*.

[29] Sermrittirong S., Van Brakel W. H., Kraipui N., Traithip S., Bunders-aelen J. (2015). Comparing the perception of community members towards leprosy and tuberculosis stigmatisation. *Leprosy Review*.

[30] WHO (2008). Advocacy, communication and social mobilization for TB control: a guide to developing knowledge, attitude and practice surveys.

[31] Nur A. Y., Rohde S. J. (2008). Factors influencing delay in seeking tuberculosis treatment in Belet-Weyne district, Somalia [Internet]. http://etd.uwc.ac.za/xmlui/bitstream/handle/11394/2778/Nur_MPH_2008.pdf?sequence=1.

[32] Adane K., Spigt M., Johanna L., Noortje D., Abera S. F., Dinant G.-J. (2017). Tuberculosis knowledge, attitudes, and practices among northern Ethiopian prisoners: implications for TB control efforts. *PLoS One*.

[33] Tolossa D., Medhin G., Legesse M. (2014). Community knowledge, attitude, and practices towards tuberculosis in Shinile town, Somali regional state, eastern Ethiopia: a cross-sectional study. *BMC Public Health*.

[34] Solliman M. A., Hassali M. A., Al-Haddad M. (2012). Assessment of knowledge towards tuberculosis among general population in North East Libya. *Journal of Applied Pharmaceutical Science*.

[35] Kalu O. O., Jimmy E. E. (2015). Assessment of knowledge, attitude and tuberculosis-related social stigma among school adolescent in a semi-urban town in Cross River State, Nigeria. *International Journal of Education and research*.

[36] Onyeonoro U. U., Chukwu J. N., Oshi D. C., Nwafor C. C., Meka A. O. (2014). Assessment of tuberculosis-related knowledge, attitudes and practices in Enugu, South East Nigeria. *Journal of infectious Diseases and Immunity*.

[37] Tobin E. A., Okojie P.-W., Isah E. C. (2013). Community knowledge and attitude to pulmonary tuberculosis in rural Edo state, Nigeria. *Annals of African Medicine*.

[38] Abioye I. A., Omotayo M. O., Alakija W. (2011). Socio-demographic determinants of stigma among patients with pulmonary tuberculosis in Lagos, Nigeria. *African Health Sciences*.

[39] Atre S., Kudale A., Morankar S., Gosoniu D., Weiss M. G. (2011). Gender and community views of stigma and tuberculosis in rural Maharashtra, India. *Glob Public Health*.

[40] Abebe G., Deribew A., Apers L. (2010). Knowledge, health seeking behavior and perceived stigma towards tuberculosis among tuberculosis suspects in a rural community in southwest Ethiopia. *PLoS One*.

[41] Mushtaq M. U., Shahid U., Abdullah H. M. (2011). Urban-rural inequities in knowledge, attitudes and practices regarding tuberculosis in two districts of Pakistan’s Punjab province. *International journal for Equity in Health*.

[42] Hatherall B., Newell J. N., Emmel N., Baral S. C., Khan M. A. (2019). "Who will marry a diseased girl?" Marriage, gender, and tuberculosis stigma in Asia. *Qualitative Health Research*.

[43] Weiss M. G., Somma D., Karim F. (2008). Cultural epidemiology of TB with reference to gender in Bangladesh, India and Malawi [Special section on gender and TB]. *The International Journal of Tuberculosis and Lung Disease*.

[44] Barter D. M., Agboola S. O., Murray M. B., Bärnighausen T. (2012). Tuberculosis and poverty: the contribution of patient costs in sub-Saharan Africa – a systematic review. *BMC Public Health*.

[45] Sima B. T., Belachew T., Abebe F. (2017). Knowledge, attitude and perceived stigma towards tuberculosis among pastoralists; do they differ from sedentary communities? A comparative cross-sectional study. *PLoS One*.

[46] Bensalah N., Hsairi M., Snene H. (2017). Community knowledge, attitude, and practices towards tuberculosis in Tunisia. *European Respiratory Journal*.

[47] (2014). *The National Strategic Plan for Tuberculosis Control 2015-2020: Towards Universal Access to Prevention, Diagnosis and Treatment*.

